# Supplementary material for: Causal Relationship between Aging and Anorexia Nervosa: A White-Matter-Microstructure-Mediated Mendelian Randomization Analysis
Source: Biomedicines. 2024 Aug 16;12(8):1874. doi: 10.3390/biomedicines12081874 (PMC11351342; doi:10.3390/biomedicines12081874)
Supplement: Supplementary file 1 [file biomedicines-12-01874-s001.zip › Supplementary Figure/Figure S4/Singe SNP plot of MR analysis for TL to ubm-b-2011.pdf]

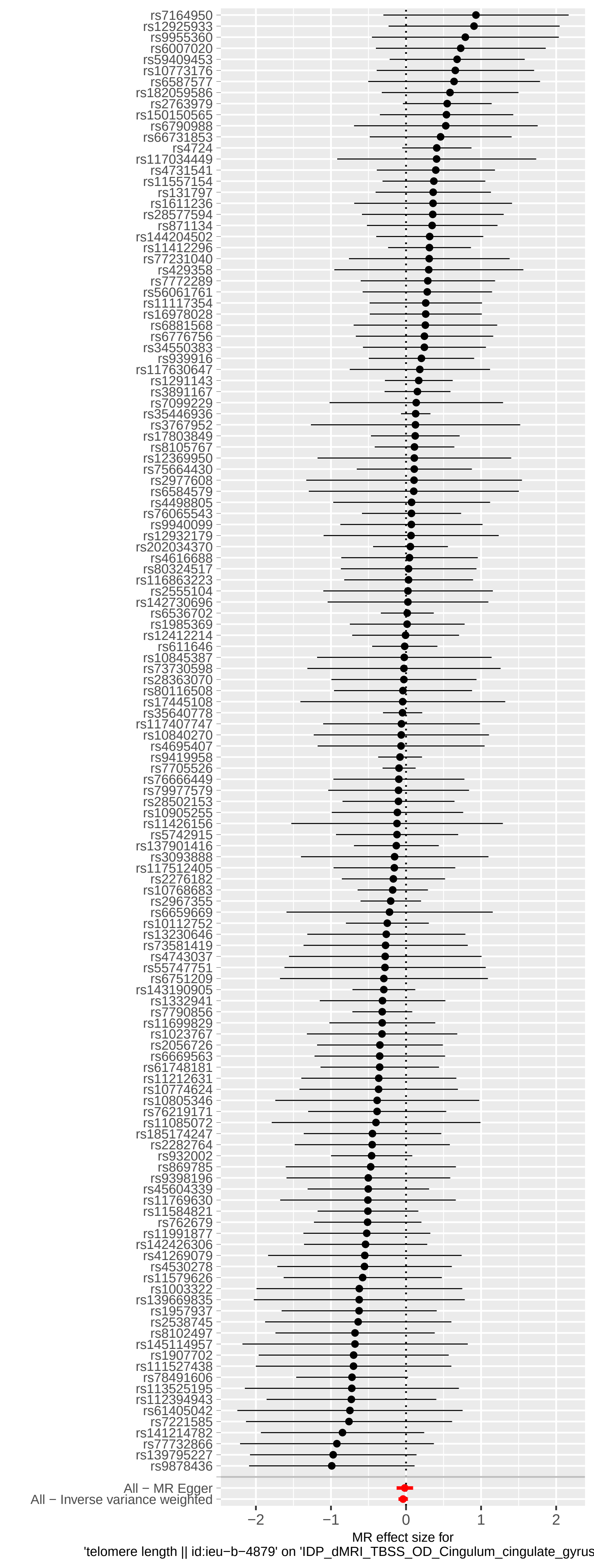
rs7164950
rs12925933
rs9955360
rs6007020
rs59409453
rs10773176
rs6587577
rs182059586
rs2763979
rs150150565
rs6790988
rs66731853
rs4724
rs117034449
rs4731541
rs11557154
rs131797
rs1611236
rs28577594
rs871134
rs144204502
rs11412296
rs77231040
rs429358
rs7772289
rs56061761
rs11117354
rs16978028
rs6881568
rs6776756
rs34550383
rs939916
rs117630647
rs1291143
rs3891167
rs7099229
rs35446936
rs3767952
rs17803849
rs8105767
rs12369950
rs75664430
rs2977608
rs6584579
rs4498805
rs76065543
rs9940099
rs12932179
rs202034370
rs4616688
rs80324517
rs116863223
rs2555104
rs142730696
rs6536702
rs1985369
rs12412214
rs611646
rs10845387
rs73730598
rs28363070
rs80116508
rs17445108
rs35640778
rs117407747
rs10840270
rs4695407
rs9419958
rs7705526
rs76666449
rs79977579
rs28502153
rs10905255
rs11426156
rs5742915
rs137901416
rs3093888
rs117512405
rs2276182
rs10768683
rs2967355
rs6659669
rs10112752
rs13230646
rs73581419
rs4743037
rs55747751
rs6751209
rs143190905
rs1332941
rs7790856
rs11699829
rs1023767
rs2056726
rs6669563
rs61748181
rs11212631
rs10774624
rs10805346
rs76219171
rs11085072
rs185174247
rs2282764
rs932002
rs869785
rs9398196
rs45604339
rs11769630
rs11584821
rs762679
rs11991877
rs142426306
rs41269079
rs4530278
rs11579626
rs1003322
rs139669835
rs1957937
rs2538745
rs8102497
rs145114957
rs1907702
rs111527438
rs78491606
rs113525195
rs112394943
rs61405042
rs7221585
rs141214782
rs77732866
rs139795227
rs9878436
All − MR Egger
All − Inverse variance weighted
−2
−1
0
1
2
MR effect size for
'telomere length || id:ieu−b−4879' on 'IDP_dMRI_TBSS_OD_Cingulum_cingulate_gyrus
